# Metastatic Melanoma Epidural Tumour Regression Following Percutaneous Radiofrequency Ablation

**DOI:** 10.7759/cureus.8039

**Published:** 2020-05-09

**Authors:** Joseph Papanikitas, Rory Fairhead, Ameen Chekroud, Siok Li Chung, David McKean

**Affiliations:** 1 Radiology, Buckinghamshire Healthcare NHS Trust, Aylesbury, GBR; 2 Radiology, Oxford University Hospitals NHS Trust, Oxford, GBR

**Keywords:** bone metastases, interventional radiology, spinal metastases, back pain, radiofrequency ablation, musculoskeletal radiology, malignant melanoma metastasis

## Abstract

Percutaneous radiofrequency ablation of spinal metastases is an emerging treatment for patients with painful metastatic spine disease. It is typically performed for patients who have not responded to conventional treatments or who have contraindications to radiotherapy. Destruction of the posterior wall of the vertebral body and epidural disease may be considered relative contraindications for radiofrequency ablation. This is due to the difficulty in achieving satisfactory ablation balanced against the risk of neural injury. We describe a case of metastatic melanoma with an expansile lytic metastasis and epidural tumour extension at the L4 vertebral body level resulting in severe central canal stenosis. This was successfully treated with percutaneous radiofrequency ablation and vertebral augmentation. The patient reported significant pain relief post-procedure and follow-up MRI at two months demonstrated local tumour regression, epidural disease resolution and improved spinal canal dimensions. To the best of our knowledge, this is the first report of epidural disease resolution from metastatic melanoma following radiofrequency ablation and emphasises the potential benefits of ablation therapy even in the presence of canal stenosis and epidural metastatic disease.

## Introduction

The spine accounts for approximately 70% of all bone metastases [1,2]. Spinal metastases are often symptomatic and may significantly impair patients' quality of life. They are also at risk of pathological fracture and potential cord compression [3]. The mainstay of treatment for the palliative treatment of painful spinal metastases is radiotherapy and analgesic drugs; however, a significant number of patients do not obtain effective pain relief from these conventional treatments. Percutaneous radiofrequency ablation has been reported to be an effective and safe treatment for spinal metastases. Current low-power bipolar systems create small and predictable ablation zones allowing for thermal treatment within the vertebra while minimising the risk of iatrogenic neural injury [4]. However, permanent neurological injury has been previously reported, including lower limb paralysis [5]. The risk of thermal damage to neurological structures may be higher if there is erosion of the posterior wall of the vertebral body as the cortex may help to contain thermal energy. As such, when ablating near the posterior wall use of an epidural thermocouple is advised to allow for real-time temperature evaluation [6,7]. Epidural metastases are less common, occurring in 5%-10% of cancer patients [8]. However, metastatic epidural disease extension is generally considered to be a relative contraindication to spinal ablation given its proximity to neural structures. This case describes successful treatment of painful melanoma metastasis in the lumbar spine with reduced tumour volume, resolution of epidural disease and improved canal dimensions [9].

## Case presentation

**Figure 1:**
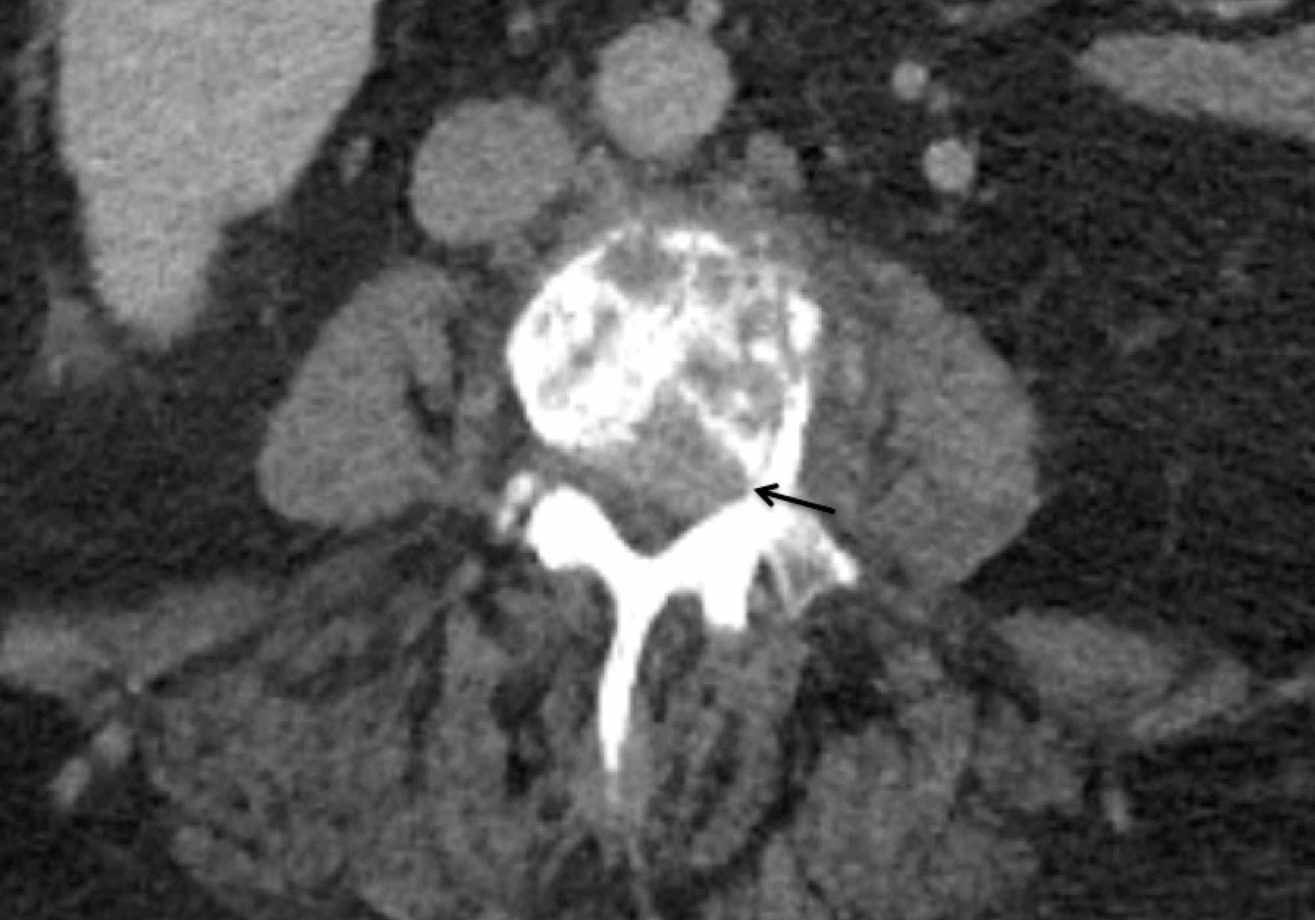
Pre-treatment portal venous phase CT demonstrating a destructive lesion within the L4 vertebral body with enhancing epidural soft tissue tumour extension (black arrow) resulting in moderate central canal stenosis.

A 73-year-old female patient presented with a history of back pain following a fall. CT demonstrated a pathological fracture of T8 and severe central canal stenosis secondary to an enhancing expansile metastasis within the L4 vertebral body (Figure 1).

**Figure 2:**
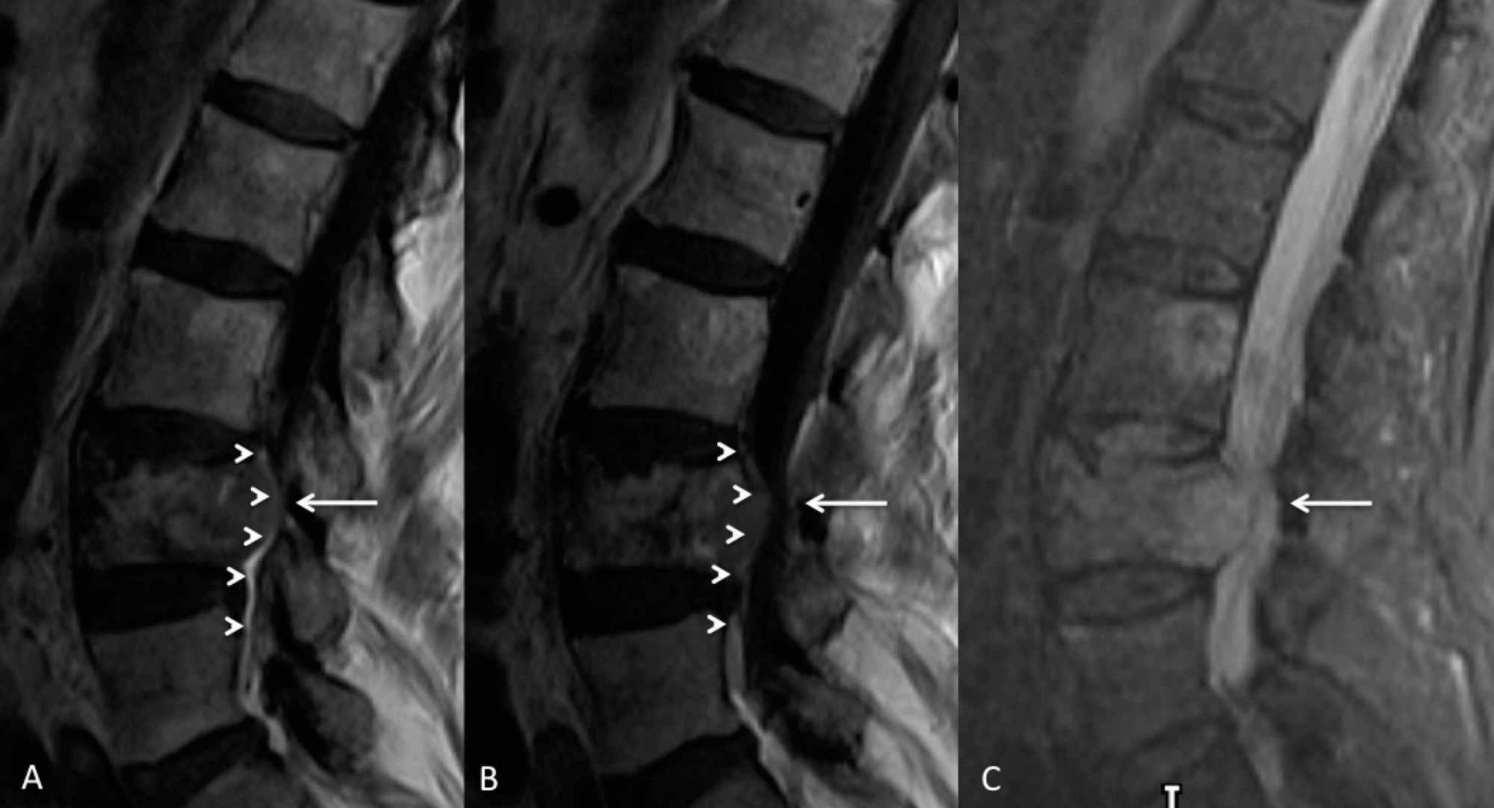
Pre-treatment parasagittal contrast-enhanced T1-weighted image (A), T1-weighted (B) and STIR-weighted (C) acquisitions demonstrating an expansile metastasis within the diffusely infiltrated L4 vertebral body and epidural metastatic disease (arrow heads) which result in moderately severe central canal stenosis (white arrow). STIR, short TI inversion recovery

**Figure 3:**
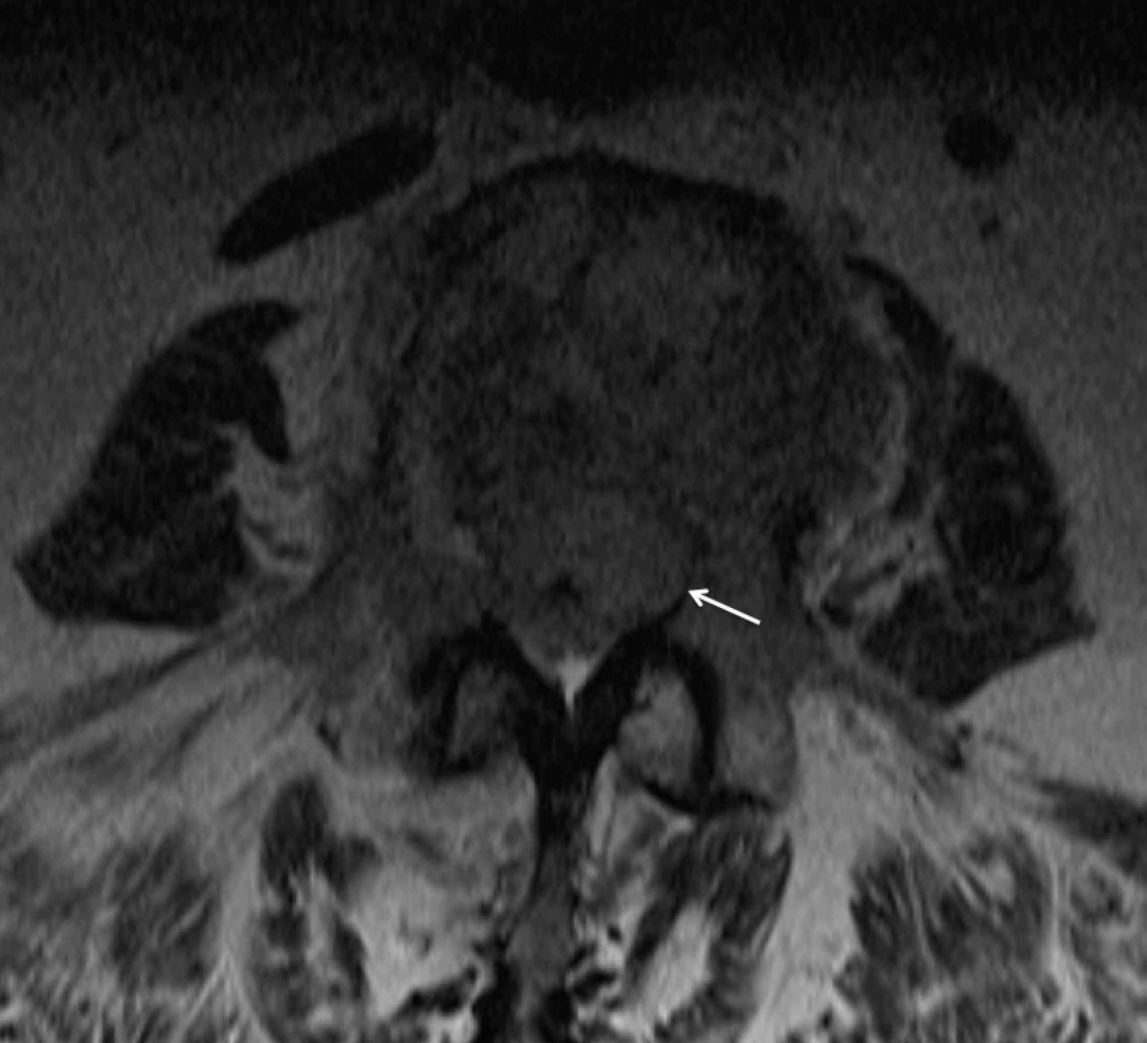
Axial T2-weighted image demonstrating a soft tissue mass extending into the epidural space (white arrow) that results in moderate central canal stenosis.

MRI revealed disseminated skeletal metastases and epidural disease extension from the L4 vertebral body metastatic deposit (Figures 2, 3).

**Figure 4:**
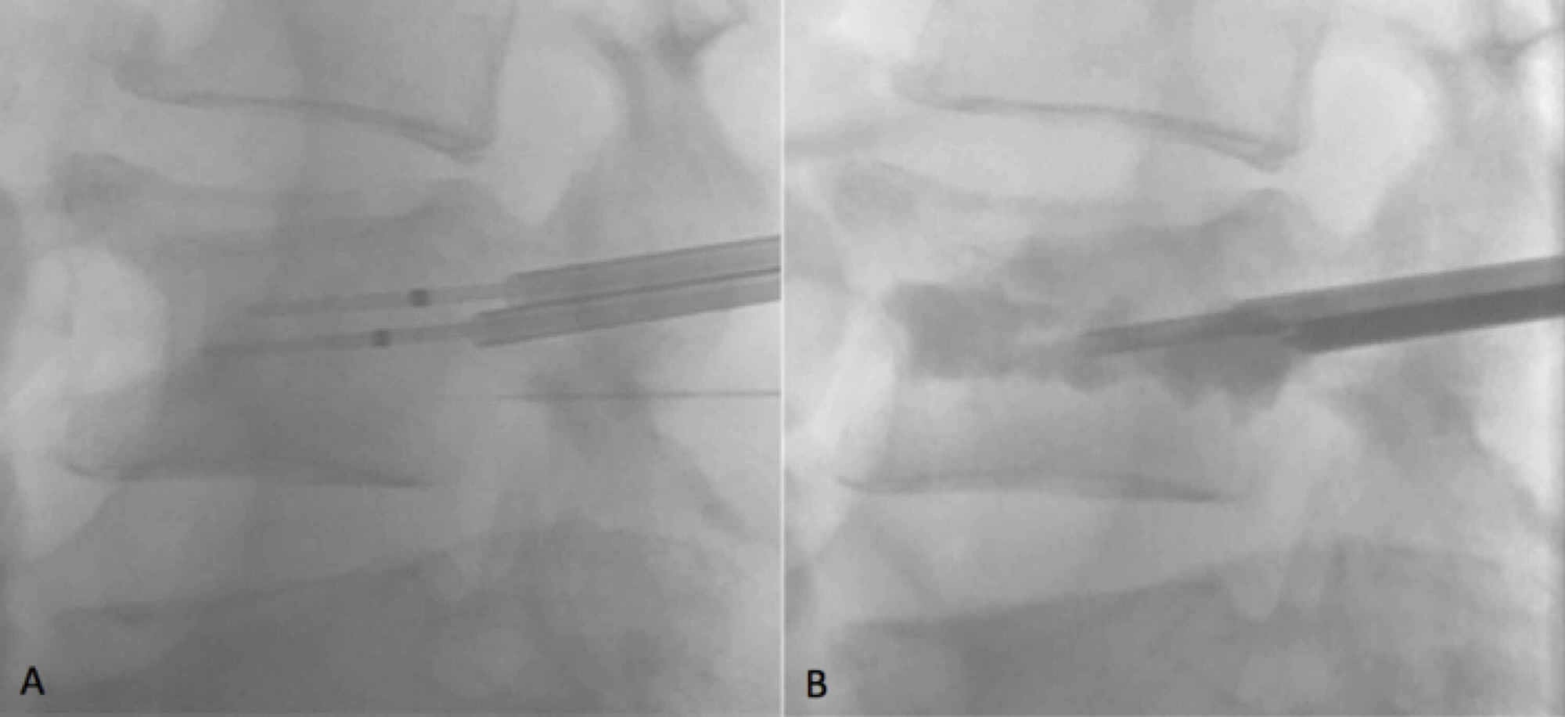
Lateral fluoroscopic images demonstrating bipedicular ablation needles and thermocouple position (A) and vertebral augmentation with methyl methacrylate (B).

Biopsy confirmed the diagnosis of metastatic melanoma, and the patient was referred for consideration of percutaneous radiofrequency ablation of the L4 lesion for palliation of the patient's symptoms of severe lower lumbar back pain. The radiofrequency ablation procedure was performed under general anaesthesia using fluoroscopic image guidance. Bipedicular 10-gauge bone access needles were advanced at the L4 vertebral level, and a bone drill was used to achieve satisfactory ablation probe position within the vertebral body (Figure 4).

**Figure 5:**
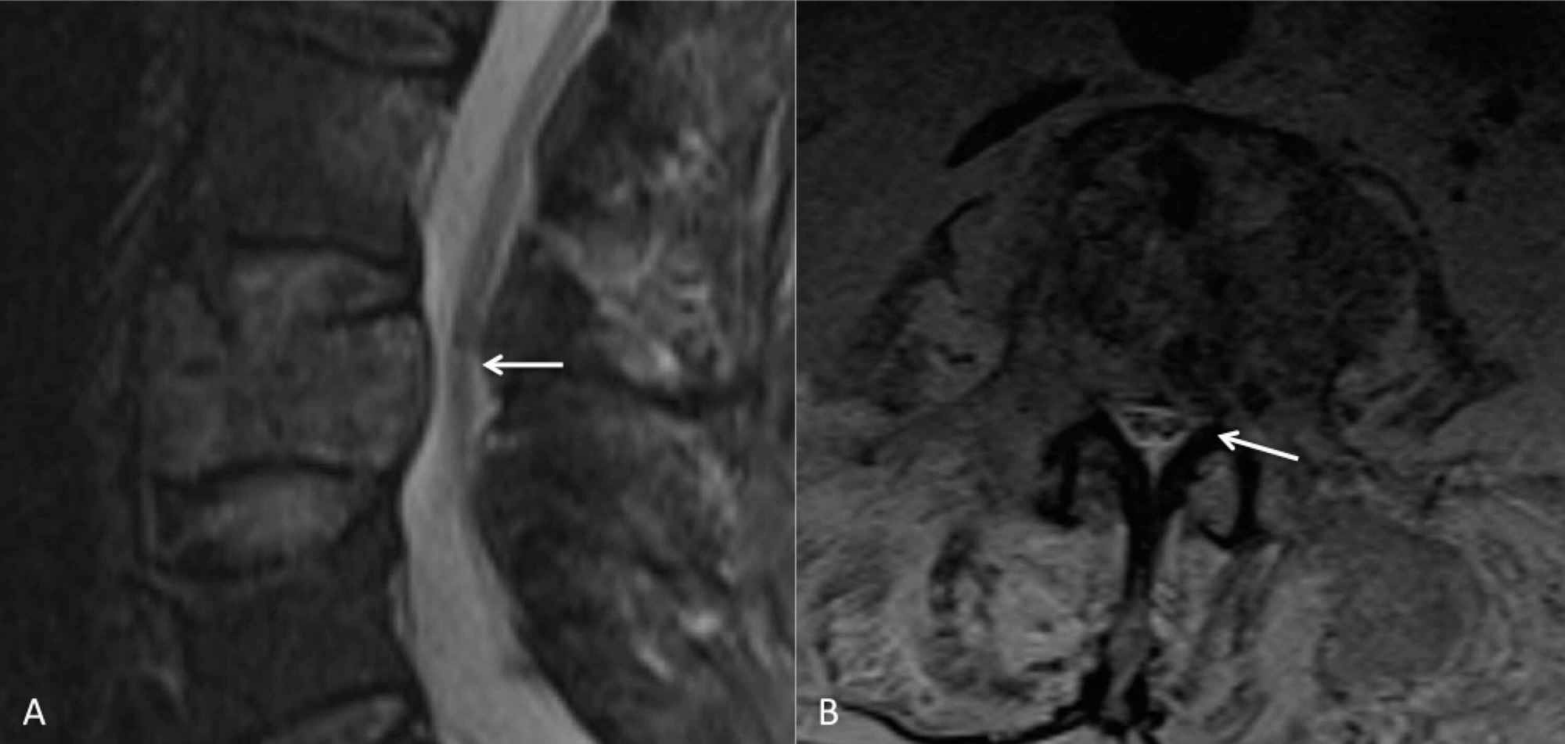
Post-treatment sagittal STIR-weighted (A) and axial T2-weighted (B) images demonstrating reduced tumour volume, resolution of epidural disease and improved canal dimensions (white arrow). STIR, short TI inversion recovery

Bipolar 17-gauge, 20-mm ablation probes were advanced, with each probe creating an ablation zone of approximately 29 mm x 21 mm. Care was taken to position the ablation probes such that the ablation zone did not extend beyond the posterior margin of the tumour, and a 20-gauge thermocouple was advanced via a transforaminal approach into the anterior epidural space to allow for continuous temperature monitoring to minimise the risk of neural injury during the ablation procedure. Following this, methyl methacrylate was injected into the L4 ablation cavity to augment the vertebral body and maintain stability. The procedure was well tolerated, and there were no immediate complications. No post-procedure neural deficit was elicited, and the patient was discharged home the same day. The patient reported significant improvement in pain at follow-up with his oncology team with reduced opiate medication requirement. The patient received no other treatment for his metastatic disease. Subsequent MRI at two months post-procedure demonstrated significant tumour regression at the L4 level with resolution of the epidural disease and improved canal dimension (Figure 5).

The patient had no recurrence of their low back pain. Unfortunately, there was disease progression elsewhere and the patient died the following month following a pulmonary emboli.

## Discussion

The spine is the most common site of osseous metastases, estimated to occur in up to 40% of cancer patients [10]. This is thought to be due to its high vascularity with seeding through the valveless extradural Batson’s venous plexus [11]. These spinal metastases may often cause severe pain, pathological fractures and spinal cord compression. Treatment for these patients is aimed at maximising pain reduction, maintaining mechanical stability and improving quality of life. Radiofrequency ablation allows for targeted destruction of the metastatic deposit and vertebral augmentation allows for stabilisation of the vertebra prevent future pathological fracture. Several studies have prove percutaneous radiofrequency ablation to be a safe technique which can be extremely effective at achieving analgesia in patients with painful spinal metastases [12]. Most recent studies have combined radiofrequency ablation with vertebral augmentation [12-15]. This provides an advantage over conventional radiotherapy alone, which may provide pain relief but offers no protection against vertebral body collapse. However, some studies have suggested that the combination of conventional radiotherapy, spinal ablation and vertebral augmentation results in higher rates of complete and overall pain response when compared with radiotherapy alone [13,14,16].

Epidural disease may be viewed as a relative contraindication to percutaneous radiofrequency spinal ablation as this has not been commonly reported to respond to ablative techniques. Conventional treatment for metastatic epidural cord compression is radiotherapy with surgery usually reserved for the patients with optimal performance status, single level epidural disease or evidence of mechanical instability [17]. One previous study has reported resolution of epidural disease secondary to prostate cancer following spinal ablation [9]. Our report is the first, to the author's knowledge, of epidural disease secondary to metastatic melanoma to respond to spinal ablation. This adds further evidence that epidural disease should not be viewed as an absolute contraindication to consideration of percutaneous radiofrequency ablation treatment. Post-procedure MRI is not performed in all cases of spinal radiofrequency ablation and as such, the true incidence of epidural response to spinal ablation is unknown. Further research is required to identify tumour types and metastasis location and morphology which may be more responsive to ablation therapy.

## Conclusions

Radiofrequency ablation is an emerging technique for the treatment of painful spinal metastases. These spinal lesions are a common finding in many cancers often extremely debilitating due to severe pain, pathological fracture and the risk of neurological compression. We report a rare case of melanoma osseous metastasis with epidural extension treated with percutaneous radiofrequency ablation with post-procedure reduced tumour volume and resolution of epidural disease. This case highlights the potential for epidural disease to respond to spinal ablation and also the potential for thermal energy diffusion beyond the vertebral body. Neural protective measures, such as the routine deployment of thermocouples to monitor temperature rise in critical areas, are advised to minimise the risk of iatrogenic injury.
